# A Numerical Study of Quantum Entropy and Information in the Wigner–Fokker–Planck Equation for Open Quantum Systems

**DOI:** 10.3390/e26030263

**Published:** 2024-03-14

**Authors:** Arash Edrisi, Hamza Patwa, Jose A. Morales Escalante

**Affiliations:** 1 Department of Physics & Astronomy, University of Texas at San Antonio, San Antonio, TX 78249, USA; arash.edrisi@my.utsa.edu (A.E.); hamza.patwa@my.utsa.edu (H.P.); 2 Department of Mathematics, University of Texas at San Antonio, San Antonio, TX 78249, USA

**Keywords:** kinetic theory, quantum information, Wigner–Fokker–Planck, Monte Carlo, Euler–Maruyama, open quantum systems, Wehrl entropy, quantum entropy, Husimi transform

## Abstract

Kinetic theory provides modeling of open quantum systems subject to Markovian noise via the Wigner–Fokker–Planck equation, which is an alternate of the Lindblad master equation setting, having the advantage of great physical intuition as it is the quantum equivalent of the classical phase space description. We perform a numerical inspection of the Wehrl entropy for the benchmark problem of a harmonic potential, since the existence of a steady state and its analytical formula have been proven theoretically in this case. When there is friction in the noise terms, no theoretical results on the monotonicity of absolute entropy are available. We provide numerical results of the time evolution of the entropy in the case with friction using a stochastic (Euler–Maruyama-based Monte Carlo) numerical solver. For all the chosen initial conditions studied (all of them Gaussian states), up to the inherent numerical error of the method, one cannot disregard the possibility of monotonic behavior even in the case under study, where the noise includes friction terms.

## 1. Introduction

Open quantum systems is an area of great importance in both Computational and Applied Mathematics and Physics due to the relevance of its applications in topics such as quantum optics, semiconductors, and lately, quantum computing and information science. In particular, quantum computing and information sciences are experiencing a booming development given the recent advances (circa 2019 by Google and IBM) in the experimental implementation of quantum computers of the order of 100 qubits in the current NISQ (Noisy Intermediate Scale Quantum) era. This reflects the need to have a holistic understanding of quantum information and quantum entropy, particularly in the present NISQ era, since, to this day, quantum computing devices are error-prone due to the effects of environmental noise. Therefore, it is also of fundamental importance to conduct an interdisciplinary study of open quantum systems encompassing this physical phenomenon, their mathematical modeling, and a computational model of their mathematical abstraction.

There is a direct connection between quantum computing and information sciences and Wigner–Fokker–Planck models. The Wigner–Fokker–Planck equation is completely equivalent to the more common model for open quantum systems in quantum computing and information, namely the Lindblad master equation, in the case where the related variables of the system are continuous (specifically position and momentum, suitable then for a phase space formulation), as opposed to spin variables for example. Open quantum systems are fundamental to the study of quantum computing and information, because, in the NISQ era, environment noise is the fundamental challenge to the preservation of information in quantum computers. Most importantly, recent techniques [1] actually make use of environment noise to get the state indeed to the ground state, from which to then start the quantum computation. These techniques are called “ground-state preparation via Lindbladians” and have been very popular in the last year in the domain of current mathematical and computational challenges in quantum computing and information.

The purpose of this work is to perform a numerical study of the behavior of quantum entropy (as a measure of quantum information) for specific computational methods of open quantum systems models, namely, Monte Carlo stochastic solvers based on Euler–Maruyama techniques. Of the different models for open quantum systems, we particularly draw our attention to the Wigner–Fokker–Planck equation, which in the case of a harmonic potential $V\left(x\right)=x^{2}/2$ (taking units such that ¯h=m=1) is as follows:

(1) $$w_{t}+k\cdot \nabla_{x}w-x\cdot \nabla_{k}w=\nabla_{\left(x,k\right)}\cdot \left(D\nabla_{\left(x,k\right)}w\right)+\gamma \nabla_{k}\cdot \left(wk\right).$$

where w(x,k,t) is the Wigner quasi-probability density function (quasi-PDF, since it might be negative in some phase space regions) defined over a position-momentum phase space $\left(x,k\right)\in R^{2}$ at time *t*, where the momentum is formally given by p=¯hk, *D* is the diffusion matrix, and γ is a friction coefficient. This model represents a quantum phase-space picture via the quasi-PDF *w* for a small subsystem in which the Hamiltonian transport is being perturbed by Markovian noise, introduced by a larger environment through energy exchanges with the subsystem. Noise is represented in this Equation then through the diffusion operator and the friction terms. For completeness, we mention that the Wigner–Fokker–Planck equation in the general case of an arbitrary potential V(x) has the following form:

(2) $$\begin{array}{c} w_{t}+k\cdot \nabla_{x}w+\Theta \left[V\right]\left\{w\right\}=\Delta_{k}w+\nabla_{k}\cdot \left(kw\right)+\Delta_{x}w,\;\mathrm{where}\\ \Theta \left[\;V\;\right]\left\{w\right\}=-i/{\left(2\pi \right)}^{d}\int \delta V\left(x,\eta \right)\;w\left(x,k^{\prime },t\right)\;\exp\left\{i\eta \cdot \left(k-k^{\prime }\right)\right\}\;dk^{\prime }d\eta \;\;\mathrm{on}\;R^{2d}, \end{array}$$

where δV(x,η)=V(x+η/2)−V(x−η/2). This more general case that includes nonharmonic potentials can be represented through the pseudo-differential integral operator Θ[V]{w} [2] above acting on the given potential V(x). However, the harmonic case will be the focus of our numerical studies, since the Wigner–Fokker–Planck equation converges to an analytically known steady-state solution [3] for potentials of this form, which can be further interpreted as capturing the effects of decoherence due to the environment.

The current state of the research field, encompassing different kinds of numerical methods for open quantum systems, in general, can be stated in the following way. Several types of numerical methods have been used in the computational modeling of open quantum systems. Their mathematical modeling through Wigner–Fokker–Planck equations and their subsequent numerical solution via Monte Carlo stochastic simulations (in the context of Quantum Optics, for example) have been reported [4], as well as discrete velocity numerical methods for a stationary Wigner Equation [5]. It is known though that there is a natural stochastic error in the numerical solution by Monte Carlo methods, where this error will decrease as $N^{-1/2}$ by increasing the number of samples *N*. Therefore, the computational cost, inferred through the number of sample points, needs to grow quadratically to reduce the error linearly. There are previous works as well on the numerical simulation of Wigner models for quantum tunneling phenomena [6], as well as literature on operator splitting type methods for the Wigner–Poisson system [7], and also on the semi-discrete analysis of the Wigner Equation [8].

The phenomena of open quantum systems need a numerical description that reflects inherently the physics of quantum transport and noise (represented by diffusion for Markovian interactions). In Monte Carlo methods for open quantum systems models, such as the Wigner–Fokker–Planck equation [4], this is achieved with the combination of finite differences representing the transport plus the random sampling from normal distributions that model the diffusion processes (via the well-known connection between random walks and diffusion in the Feynman-Kac formulation). The combination of these two is called the Euler–Maruyama method (to first order), and it is a known numerical procedure for the solution of stochastic differential equations [9]. Extensions of the Monte Carlo method described are possible for Wigner functions that have a negative part, such as signed-particle methods [10,11]. This would be achieved by splitting the Wigner function into the sum of two parts, a positive part $w_{+}$ and a negative part $w_{-}$, both of which on their own can formally be considered probability densities. The distributions associated with $w_{+}$ and $w_{-}$ would be related: when one loses a particle, the other would gain one, and vice versa. On the other hand, Discontinuous Galerkin (DG) methods can be designed to mimic numerically convection-diffusion problems, as is the case for Local DG or Nonsymmetric Interior Penalty Galerkin methods. For example, Ref. [2] focused on an adaptable DG scheme for Wigner–Fokker–Planck, where a Nonsymmetric Interior Penalty Galerkin method was used as a numerical method. There is literature on the use of Discontinuous Galerkin Methods in equations of the Quantum–Liouville type [12], as well as numerical modeling of a Quantum Liouville–Poisson system [13]. However, the aforementioned equations in the last sentence consider only quantum transport in the problem, because diffusion does not appear in Liouvillian transport. Previous works where the noise due to the environment in an open quantum system is modeled in a DG setting for master equations have been in reported in [14,15], for example.

In the particular context of mathematical studies of entropy in open quantum systems, Ref. [3] uses classical (mathematically speaking) entropy methods from kinetic theory, which consider relative entropies:

(3) $$e_{\varphi }\left(f|g\right)=\int_{R^{n}}\varphi \left(f/g\right)g\left(dx\right),$$

(where $e_{\varphi }$ is the relative entropy function of *f* with respect to g, and φ the generating function) such as the logarithmic relative entropy, related to:

(4) $$\varphi_{1}\left(\alpha \right)=\alpha \ln\left(\alpha \right)-\alpha +1,\;\alpha \in R^{+},$$

or the quadratic relative entropy, related to:

(5) $$\varphi_{2}\left(\alpha \right)=K{\left(\alpha -1\right)}^{2},\;\alpha \in R,\;K>0,$$

to establish the conditions that imply the existence of a thermal equilibrium state, proving an exponential decay towards it. In [16], the free energy (or Kullback relative entropy of ρ with respect to exp(−V)) is presented for the study of Fokker–Planck equations. In [17], entropy methods for diffusive PDEs are presented in general, and in particular as well for Fokker–Planck equations which might be possibly nonlinear.

Though the physical entropy in quantum Mechanics for a density matrix representation, the Von Neumann entropy:

(6) $$S=-\mathrm{Tr}(\hat{\rho }\log\left(\hat{\rho }\right))$$

which is well known and constitutes the quantum extension of the classical Boltzmann/Shannon entropy:

(7) $$H=-\int_{\Omega_{x}}\int_{\Omega_{p}}f\log\left(f\right)dxdp.$$

The definition of entropy in the Wigner formulation is not as direct as an extension since log(w) might not be able to be evaluated as a real-valued function for cases where w<0 (which can happen; however, for Gaussian states, their Wigner function w(x,p,t)≥0 is nonnegative). However, one can use the Wehrl entropy [18], which is essentially the Boltzmann/Shannon *H* entropy abovementioned applied to the nonnegative Husimi transform of the Wigner function. Previous mathematical work [19] has also indicated that the only physically relevant quantum Fokker–Planck equation that makes quantum entropy grow monotonically (for all admissible initial conditions) is the frictionless one (γ=0). However, mathematical studies of the quantum relative entropy in the Wigner–Fokker–Planck equation are indeed helpful and necessary to establish the convergence of its initial value problem to the steady-state solution in the harmonic problem precisely through entropy methods [3] as abovementioned. The result provided in [3] proves mathematically the convergence to the steady state.

Naturally, the abovementioned relative entropy results in [3] are crucial for showing convergence to a steady-state solution for the Wigner–Fokker–Planck initial value problem (IVP) under a harmonic potential, via entropy methods. There will be two distinctions between the nature of this result and our numerical studies though. The first one is that we will focus on the absolute entropy, not the relative entropy, where, as we have mentioned, it is known that monotonic behavior of the physical Von Neumann entropy is only guaranteed for the frictionless case γ=0.

Second, our studies will not include the Von Neumann entropy but focus on the Wehrl entropy (absolute), where rather than the Wigner function it is the Husimi function the one in Wehrl’s entropy argument. Therefore, though there are similarities in the functional form of the relative logarithmic entropy for the Wigner function *w* (or rather their positive and negative parts $w^{\pm }$) and the absolute Wehrl entropy for the Husimi function h(w); in reality, they are analytically different. On the other hand, a generalized study of Fokker–Planck equations indicates that interpreting them as gradient flows in a Wasserstein metric (via a variational formulation of them) [20] is helpful to find exponential convergence of them by entropy production studies. Works related to this research direction have been performed in [16] and [21] for the Fokker–Planck equation, for the Lindblad equation in [22,23,24]. There is work as well on the gradient flows of the entropy for finite Markov Chains [25], as well as on the exponential decay of Rényi divergence under Fokker–Planck equations [26].

Regarding numerical studies of entropy in kinetic equations, there are previous numerical studies on kinetic equations for different applications, where the numerical entropy is monitored during the time evolution of the respective problem. Work on this regard has been done for the Vlasov–Boltzmann–Poisson system for electron transport in semiconductors [27], for the Vlasov–Poisson system [28], as well as for Fokker–Planck–Landau Type Equations for plasmas [29], and for the coupled Vlasov–Poisson Fokker–Planck–Landau equation [30], describing transport plasma models for Coulomb, Maxwell type, and hard-sphere particle interactions. In the particular application of open quantum systems, however, to the best of our knowledge, a numerical study of the absolute quantum entropy for the Wehrl entropy in the case of a Wigner–Fokker–Planck equation is lacking in the literature. Our contribution to the discipline is to fill this gap by studying precisely these numerical aspects in the particular case of a harmonic potential, which is the main aim of our work. We conclude from our numerical studies that, for most of the chosen initial conditions (all of them Gaussian states), we have observed monotonic behavior of the Wehrl entropy, whereas for one case, though the behavior might seem nonmonotonic without including the inherent error of the stochastic method used, one cannot disregard the possibility of monotonic behavior when considering the uncertainty error natural to our stochastic Monte Carlo method.

## 2. Materials and Methods

In this work, we utilize stochastic numerical methods of the Monte Carlo type (based on the Euler–Maruyama method) appropriate for the solution of the convective-diffusive Wigner–Fokker–Planck equation under a harmonic potential. Given the known initial and steady states, we compute the time evolution using the Monte-Carlo Euler–Maruyama Method, monitoring as well the $L_{2}$-type distance to the steady state and the Wehrl entropy applied to the Husimi transform as a sanity check. We solve analytically for the Husimi transform of a Gaussian state of the Wigner–Fokker–Planck, finding the expected initial and final values of the Wehrl entropy. We study two particular cases of Gaussian states: the harmonic groundstate (since we will choose it as the initial condition of our initial value problem) and the steady-state analytical solution to which we expect to converge numerically, as well as Gaussian states (Gaussian Wigner functions) whose covariance matrix is proportional to one of the two previously mentioned cases.

### 2.1. Monte-Carlo Solver: Euler–Maruyama Method

We will be using the ground state of the quantum harmonic oscillator in its Wigner representation as our initial condition in the numerical solution of the Wigner–Fokker–Planck Equation [31]:

(8) $$w_{0}\left(x,p\right)=\frac{2}{h}\;e^{-\frac{a^{2}p^{2}}{{¯\;h}^{2}}-\frac{x^{2}}{a^{2}}}.$$

To tackle the numerical solution of the Wigner–Fokker–Planck equation, we will employ the Euler–Maruyama method. This well-known stochastic numerical technique is particularly well-suited for simulating the dynamics of quantum systems in the presence of noise, namely open quantum systems. The Euler–Maruyama method is used to discretize the evolution of the Wigner function by following the trajectory of points in phase space, randomly sampling from the initial condition as a probability distribution (since it is a nonnegative function given that it is a Gaussian state, and therefore, its Wigner function is a Gaussian) to generate our sample of points constituting a point distribution. To choose successive states for our Wigner–Fokker–Planck kinetic equation in our simulations, we discretize both the transport (via a forward Euler time-step) and diffusion (via a random walk simulation of it by sampling from a Gaussian distribution with covariance related to the diffusion matrix representing the environment noise) processes over small time steps, incorporating in this way both deterministic transport and random diffusion components of the dynamics. Therefore, the phase-space point that represents the state of the system at the successive time t+Δt for the *i*-th trajectory in particular is given by:

(9) $$\left(x_{i},p_{i}\right)\left(t+\Delta t\right)=\left(x_{i},p_{i}\right)\left(t\right)+\Delta t\left(p_{i},-x_{i}-\gamma p_{i}\right)\left(t\right)+{\vec{E}}_{i},$$

where ${\vec{E}}_{i}\in R^{2}$ is a random variable sampled from a Gaussian with covariance matrix 2DΔt. More information about this is given in Section 2.2 and Equation (11). This approach allows us to mimic discretely the continuous evolution of the quantum state considering as well the random fluctuations induced by the environment. The Euler–Maruyama method is a numerical technique that falls under the category of Monte Carlo methods, specifically within the realm of stochastic differential Equations (SDEs). Monte Carlo methods involve the use of random sampling to obtain numerical results, and in the context of SDEs, these methods are employed to simulate the evolution of stochastic processes. The Euler–Maruyama method is particularly suited for solving stochastic differential equations of the form below:

(10) $$dX_{t}=a\left(X_{t}\right)dt+b\left(X_{t}\right)dW_{t},$$

where $X_{t}$ is the state of the system at time *t*, $a(X_{t})$ and $b(X_{t})$ are deterministic functions, dt is the differential time step, and $dW_{t}$ is the differential increment of a Wiener process/Brownian motion. The stochastic term $dW_{t}=W_{n+1}-W_{n}$ denotes one step in the random walk. However, we approximate this difference as 2DΔtN(0,1), where N(0,1) represents the normal distribution with a mean of zero and a variance of one [9], and we have denoted by *D* the value of the diffusion matrix in the numerical simulations as well. The specifics are explained below.

### 2.2. Pseudo-Code and Methodology Description

The Wigner–Fokker–Planck equation can be formally considered to be related to an SDE of the form (10). As abovementioned, Monte Carlo simulation methods have been proposed for solving equations of this type, in the context of quantum optics [4]. Let the Wigner function at time t=0 be w(x,p,0). We then obtain an initial collection of *N* points $\left(q_{i}\left(0\right),p_{i}\left(0\right)\right)\equiv {\vec{z}}_{i}\left(0\right)$ in phase space by randomly sampling from w(x,p,0). To each point ${\vec{z}}_{i}\left(t\right)$, a transport and diffusion part are applied to evolve the point to the next time step t+Δt, as per the Euler–Maruyama method:

(11) $${\vec{z}}_{i}\left(t+\Delta t\right)={\vec{z}}_{i}\left(t\right)+{\vec{\alpha }}_{i}\left(t\right)\Delta t+{\vec{E}}_{i},$$

where ${\vec{\alpha }}_{i}=\left(p_{i},-q_{i}-\gamma p_{i}\right)$ is the transport vector for Equation (1), and ${\vec{E}}_{i}\in R^{2}$ is a random variable with covariance matrix 2DΔt. The term with ${\vec{\alpha }}_{i}$ represents the transport process (deterministic) and the term with ${\vec{E}}_{i}$ represents the diffusion (random) process. These processes are applied iteratively to all points at each time step. At the final time *T*, we obtain a final set of points ${\vec{z}}_{i}\left(T\right)$. This set of points is equivalent to a distribution obtained by sampling *N* points from the analytical solution of the WFP equation at time *T*.

It is important to note that this method applies in principle to nonnegative Wigner functions since Monte Carlo methods such as the Euler–Maruyama algorithm use probability densities, which are nonnegative by definition. Though one can use decompositions of the Wigner function into the difference $w^{+}-w^{-}$ of two nonnegative Wigner functions $w^{+}$ and $w^{-}$ as in the so-called signed-particle method [32,33,34], we only consider in this work specific initial conditions and potentials (namely, Gaussian states and harmonic potentials, respectively) for the method of choice that have the property w(x,p,0)≥0⇒w(x,p,t)≥0∀t>0. This is because it is known that for the case when w(x,p,0) is a Gaussian state and the potential is harmonic, the time-evolution induced by the Wigner–Fokker–Planck dynamics (1) keeps the state Gaussian at all times, thus rendering a nonnegative Wigner function [35]. This justifies our use of the harmonic oscillator potential and its ground state (8) (which is a Gaussian state) as the initial condition.

The following pseudo-code (see Algorithm 1) depicts the implementation of our method (since in our units ¯h=1, we have denoted the momentum as p=¯hk).

#### Algorithm Specifics for the Monte Carlo Solver of Wigner–Fokker–Planck

1. Parameter Definitions
  - *L*: Length of the domain in position space.
  - $\sigma_{q}$ and $\sigma_{p}$: Diagonal matrix elements of the covariance matrix of the Wigner function at the initial time.
  - $\delta_{t}$: Time step for the simulation.
  - μ: Mean value vector for the initial distribution.
  - $\mu_{1}$ and $\mu_{2}$: Components of the mean value vector for the position and momentum, respectively.
  - $D_{qq}$ and $D_{pp}$: Diffusion coefficients for position and momentum, respectively.
  - *D*: Diffusion matrix (assumed having zero off-diagonal terms).
  - γ: Friction coefficient.
2. Array Definitions
  - *q*: Array to store position values for each particle at each time step.
  - *p*: Array to store momentum values for each particle at each time step.
3. Initial Conditions
  - Gaussian Sampling: Initialize the position and momentum of each particle at the first time step using normal random number generation with mean components $\mu_{1},\;\mu_{2}$ and standard deviations $\sigma_{q}$, $\sigma_{p}$.
4. Time Evolution Loop
  - Use nested loops to iterate over each time step *i* and each particle *j*.
  - Generate random noise ϵ using a multivariate normal distribution with mean μ and 2DΔt as covariance matrix for position and momentum.
  - Update the position q[i+1,j] and momentum p[i+1,j] of each particle using the Euler–Maruyama stochastic method.
5. Simulation Output
  - After the completion of the time evolution loop, the arrays *q* and *p* contain the simulated trajectories of position and momentum for each particle over time.

**Algorithm 1** Euler–Maruyama for the Wigner–Fokker–Planck equation (harmonic potential)
  1:
**Define:**
  2:

L←1.0

  3:

$\sigma_{q}\leftarrow \frac{L}{\sqrt{2}}$

  4:

$\sigma_{p}\leftarrow \frac{1}{\sqrt{2}L}$

  5:

$\delta_{t}\leftarrow 0.01$

  6:

Total_Time←50.

  7:

$NumOfTimeStep\leftarrow \mathrm{round}\{\frac{Total\_Time}{\delta_{t}}\}$

  8:

$NumOfParticles\leftarrow 10^{4}$

  9:

$\mu_{1}\leftarrow 0.$

10:

$\mu_{2}\leftarrow 0.$

11:

$\mu \leftarrow \left[\begin{array}{cc} \mu_{1} & \mu_{2} \end{array}\right]$

12:

$D_{qq}=1.,\;D_{pp}=1.$

13:

γ=1.

14:

$D\leftarrow \left[\begin{array}{cc} D_{qq} & 0\\ 0 & D_{pp} \end{array}\right]$

15:
**Arrays Initialization:**
16:

q←zeros[NumOfTimeStep,NumOfParticles]

17:

p←zeros[NumOfTimeStep,NumOfParticles]

18:
**Initial Conditions:**
19:

$q\left[1,:\right]\leftarrow \mathrm{normrnd}(\mu \left[1\right],\;\sigma_{q},\;\left[1,\;NumOfParticles\right])$

20:

$p\left[1,:\right]\leftarrow \mathrm{normrnd}(\mu \left[2\right],\;\sigma_{p},\;\left[1,\;NumOfParticles\right])$

21:
**Update:**
22:**for each** i∈NumOfTimeStep **do**23:    **for each** j∈NumOfParticles **do**24:        $\epsilon \leftarrow \mathrm{mvnrnd}(\mu ,\;2D\delta_{t})$25:        $q\left[i+1,\;j\right]\leftarrow q\left[i,j\right]+p\left[i,j\right]\delta_{t}+\epsilon \left[1\right]$26:        $p\left[i+1,\;j\right]\leftarrow p\left[i,j\right]+\left(-q[i,j]-\gamma p[i,j]\right)\delta_{t}+\epsilon \left[2\right]$27:    **end for**28:
**end for**



The algorithm simulates the stochastic evolution of a system governed by a Langevin equation, incorporating random noise to account for the effects of an external environment. This type of simulation is commonly used in the study of open quantum systems, where the Euler–Maruyama method provides a computationally efficient approach to capture the stochastic dynamics of the system. The generated trajectories allow researchers to analyze the behavior of the system and study phenomena, such as decoherence and dissipation in quantum systems. Langevin dynamics is a mathematical and computational framework used to model the motion of particles in a physical system subject to random forces and friction. It is commonly applied in various scientific fields, including physics, chemistry, and biology, to describe the stochastic behavior of particles in a medium. The Langevin equation, named after the French physicist Paul Langevin, is a stochastic differential equation that incorporates both deterministic and random components to simulate the dynamics of a particle. The equation is often used in the context of Brownian motion, where particles experience random forces due to collisions with surrounding molecules or other particles.

We have written a code, in MATLAB and also its equivalent version in Python, both available in the GitHub repository https://github.com/phjame/StochasticWFP (accessed on 29 February 2024), for the computational implementation of our Euler–Maruyama-based Monte Carlo solver. For both languages, we have used their respective MATLAB and Python statistical toolboxes to fit a 2D Gaussian distribution onto each set of phase-space points for every time step. Subsequently, we utilize the resulting covariance matrix at each time step to compute the respective Wehrl quantum entropy, as well as to monitor the $L_{2}$-norm between our numerical Wigner function at each time step and the steady-state solution μ:

(12) $$\mu \left(x,k\right)=\frac{1}{2\pi \sqrt{5}}\;e^{-(\frac{1}{5}|x{|}^{2}+\frac{1}{5}x\cdot k+\frac{3}{10}|k{|}^{2})}$$

The inequality condition for $L_{2}$-norm [2] is as follows:

(13) $$\;{\left||\frac{w-\mu }{\sqrt{\mu }}|\right|}_{L^{2}\left(R^{2d}\right)}\leq e^{-\sigma t}{\left||\frac{w_{I}-\mu }{\sqrt{\mu }}|\right|}_{L^{2}\left(R^{2d}\right)}\;$$

with σ such that the Hessian of the quadratic form inside the argument of the Gaussian representing the steady-state solution satisfies:

(14) $$\;Hess\left(\frac{1}{5}{\left|x\right|}^{2}+\frac{1}{5}x\cdot k+\frac{3}{10}{\left|k\right|}^{2}\right)\geq \sigma I.$$

More about these norm considerations can be found in Appendix A.

## 3. Results

### 3.1. Wigner–Fokker–Planck Model: Gaussian States under Harmonic Potential

#### 3.1.1. Husimi Transform of a Gaussian State (Gaussian Wigner Function)

We want to find the Husimi transform of a Gaussian state, given by the Wigner function:

$$w\left(x,k\right)=\frac{1}{\sqrt{{|\Sigma |\left(2\pi \right)}^{d}}}\exp\left(-\frac{1}{2}\left(x,k\right)\Sigma^{-1}\left(\begin{array}{c} x\\ k \end{array}\right)\right)=\frac{1}{2\pi \sqrt{\left|\Sigma \right|}}\exp\left(-\frac{1}{2}\left(x,k\right)\Sigma^{-1}\left(\begin{array}{c} x\\ k \end{array}\right)\right)$$

or in a position-momentum representation, since p=¯hk:

$$w\left(x,p\right)=\frac{1}{2\pi \sqrt{\left|\Sigma \right|}}\exp\left(-\frac{1}{2}\left(x,p/¯\;h\right)\Sigma^{-1}\left(\begin{array}{c} x\\ p/¯\;h \end{array}\right)\right)$$

To calculate the Wehrl entropy:

H=−∫hlog(h)dxdp,

we need to calculate the Husimi function, obtained by applying the Husimi transform to our Wigner function above. Namely, we have:

$$h\left(x,p\right)=\int \int w\left(x^{\prime },p^{\prime }\right){\left(\pi ¯\;h\right)}^{-1}\exp\left(-{\left(x^{\prime }-x\right)}^{2}/2s^{2}\right)\exp\left(-{\left(p^{\prime }-p\right)}^{2}\left(2s^{2}/{¯\;h}^{2}\right)\right)dx^{\prime }dp^{\prime }.$$

Thus, concretely, we have that the Husimi function for a Gaussian state is:

$$h=\frac{{\left(\pi ¯\;h\right)}^{-1}}{2\pi \sqrt{\left|\Sigma \right|}}\int \int \exp\left(-\frac{\left(x^{\prime },\frac{p^{\prime }}{¯\;h}\right)}{2}\Sigma^{-1}\left(\begin{array}{c} x^{\prime }\\ \frac{p^{\prime }}{¯\;h} \end{array}\right)\right)\exp\left(-\frac{{\left(x^{\prime }-x\right)}^{2}}{2s^{2}}\right)\exp\left(-\frac{{\left(p^{\prime }-p\right)}^{2}2s^{2}}{{¯\;h}^{2}}\right)dx^{\prime }dp^{\prime },$$

or if we stick with wave-numbers *k* instead of momentum, we have:

$$h\left(x,k\right)=\frac{{\left(\pi ¯\;h\right)}^{-1}¯\;h}{2\pi \sqrt{\left|\Sigma \right|}}\int \int \exp\left(-\frac{\left(x^{\prime },k^{\prime }\right)}{2}\Sigma^{-1}\left(\begin{array}{c} x^{\prime }\\ k^{\prime } \end{array}\right)\right)\exp\left(-\frac{{\left(x^{\prime }-x\right)}^{2}}{2s^{2}}\right)\exp\left(-2s^{2}{\left(k^{\prime }-k\right)}^{2}\right)dx^{\prime }dk^{\prime },$$

equivalent to:

$$h\left(x,k\right)=\frac{\int \int \exp\left(-\frac{\left(x^{\prime },k^{\prime }\right)}{2}\Sigma^{-1}\left(\begin{array}{c} x^{\prime }\\ k^{\prime } \end{array}\right)\right)\exp\left(-\frac{\left(x^{\prime }-x,k^{\prime }-k\right)}{2}\left(\begin{array}{cc} \frac{1}{s^{2}} & 0\\ 0 & 4s^{2} \end{array}\right)\left(\begin{array}{c} x^{\prime }-x\\ k^{\prime }-k \end{array}\right)\right)dx^{\prime }dk^{\prime }}{2\pi^{2}\sqrt{\left|\Sigma \right|}}.$$

If we use the notation:

$$\Sigma^{-1}=\left(\begin{array}{cc} \Sigma_{11}^{-1} & \Sigma_{12}^{-1}\\ \Sigma_{21}^{-1} & \Sigma_{22}^{-1} \end{array}\right)=\left(\begin{array}{cc} a & b\\ c & d \end{array}\right)$$

then one can prove that:

$$h=\frac{1}{\pi \sqrt{\left|\Sigma \right|}}\frac{\exp(\frac{\frac{c^{2}k^{2}}{2}s^{4}-2dk^{2}s^{2}\left(1+as^{2}\right)-2cks^{2}x+\frac{c^{2}x^{2}}{8}-adx^{2}/2-2as^{2}x^{2}+b^{2}\left(\frac{k^{2}s^{4}}{2}+\frac{x^{2}}{8}\right)+b\left(ck^{2}s^{4}-2ks^{2}x+\frac{cx^{2}}{4}\right)}{d\left(1+as^{2}\right)+s^{2}\left(4-\frac{{\left(b+c\right)}^{2}}{4}+4as^{2}\right)})}{\sqrt{4-\frac{{\left(b+c\right)}^{2}}{4}+4as^{2}+ad+d/s^{2}}}.$$

Furthermore, considering that the covariance matrix is positive semi-definite and, therefore, symmetric, then we have $\Sigma_{12}^{-1}=b=c=\Sigma_{21}^{-1}$, so:

$$h\left(x,k\right)=\frac{1}{\pi }\frac{\sqrt{|\Sigma^{-1}|}\exp\left(\frac{-2s^{2}\left(1+as^{2}\right)dk^{2}-4bs^{2}kx-\frac{1}{2}a\left(d+4s^{2}\right)x^{2}+\frac{1}{2}b^{2}\left(4k^{2}s^{4}+x^{2}\right)}{d\left(1+as^{2}\right)+s^{2}\left(4-b^{2}+4as^{2}\right)}\right)}{\sqrt{4-b^{2}+a\left(4s^{2}+d\right)+\frac{d}{s^{2}}}},$$

which is equivalent to:

$$h\left(x,k\right)=\frac{1}{\pi }\frac{\sqrt{|\Sigma^{-1}|}}{\sqrt{|\Sigma^{-1}|+4\left(1+as^{2}\right)+\frac{d}{s^{2}}}}\exp\left(-\frac{1}{2}\frac{\left(\frac{|\Sigma^{-1}|}{s^{2}}-4a\right)x^{2}+8bkx+4(d+|\Sigma^{-1}\left|s^{2}\right)k^{2}}{\left[|\Sigma^{-1}|+4\left(1+as^{2}\right)+\frac{d}{s^{2}}\right]}\right)$$

or in a component-wise form:

$$h\left(x,k\right)=\frac{1}{\pi }\frac{\sqrt{|ad-b^{2}|}\exp\left(\frac{\left[b^{2}-a\left(d+4s^{2}\right)\right]x^{2}-8bs^{2}kx+4s^{2}\left[b^{2}s^{2}-\left(1+as^{2}\right)d\right]k^{2}}{2[d\left(1+as^{2}\right)+s^{2}\left(4-b^{2}+4as^{2}\right)]}\right)}{\sqrt{4-b^{2}+a\left(4s^{2}+d\right)+\frac{d}{s^{2}}}}$$

which we can also write as:

$$h\left(x,k\right)=\frac{1}{\pi }\frac{\sqrt{|ad-b^{2}|}}{\sqrt{4-b^{2}+a\left(4s^{2}+d\right)+\frac{d}{s^{2}}}}\exp\left(-\frac{1}{2}\left(x,k\right)S^{-1}\left(\begin{array}{c} x\\ k \end{array}\right)\right)$$

with the new inverse covariance matrix:

$$S^{-1}=\frac{1}{d\left(1+as^{2}\right)+s^{2}\left(4\left[1+as^{2}\right]-b^{2}\right)}\left(\begin{array}{cc} a\left(d+4s^{2}\right)-b^{2} & 4bs^{2}\\ 4bs^{2} & 4s^{2}\left[\left(1+as^{2}\right)d-b^{2}s^{2}\right] \end{array}\right).$$

The covariance matrix *S* is the one we will use in our calculations of the Wehrl entropy for the Husimi function of a Gaussian state.

#### 3.1.2. Husimi Transform of Steady-State Solution for Harmonic Benchmark Problem

We only need to find specifically the covariance matrix for this case. Since we have in general that:

$$S^{-1}=\frac{1}{d\left(1+as^{2}\right)+s^{2}\left(4\left[1+as^{2}\right]-b^{2}\right)}\left(\begin{array}{cc} a\left(d+4s^{2}\right)-b^{2} & 4bs^{2}\\ 4bs^{2} & 4s^{2}\left[\left(1+as^{2}\right)d-b^{2}s^{2}\right] \end{array}\right)$$

where:

$$\Sigma^{-1}=\left(\begin{array}{cc} \Sigma_{11}^{-1} & \Sigma_{12}^{-1}\\ \Sigma_{21}^{-1} & \Sigma_{22}^{-1} \end{array}\right)=\left(\begin{array}{cc} a & b\\ c & d \end{array}\right),$$

so for the inverse covariance matrix of the steady-state solution, we have:

$$\Sigma_{\infty }^{-1}=\left(\begin{array}{cc} 2/5 & 1/5\\ 1/5 & 3/5 \end{array}\right)=\left(\begin{array}{cc} 0.4 & 0.2\\ 0.2 & 0.6 \end{array}\right),$$

since:

$$\Sigma_{\infty }=\left(\begin{array}{cc} 3 & -1\\ -1 & 2 \end{array}\right).$$

Choosing the parameter value $s=1/\sqrt{2}\Leftrightarrow 2s^{2}=1,$ then we have:

$$S_{\infty }^{-1}=\frac{1}{d+a+ad/2-b^{2}/2+2}\left(\begin{array}{cc} a\left(d+2\right)-b^{2} & 2b\\ 2b & \left[\left(2+a\right)d-b^{2}\right] \end{array}\right)$$

$$S_{\infty }^{-1}=\frac{1}{\frac{3}{5}+\frac{2}{5}+\frac{2}{5}\frac{3}{5}/2-{\frac{1}{5}}^{2}/2+2}\left(\begin{array}{cc} \frac{2}{5}\left(\frac{3}{5}+2\right)-{\frac{1}{5}}^{2} & 2\frac{1}{5}\\ 2\frac{1}{5} & \left[\left(2+\frac{2}{5}\right)\frac{3}{5}-{\frac{1}{5}}^{2}\right] \end{array}\right)$$

$$S_{\infty }^{-1}=\frac{1}{1+\frac{1}{10}+2}\left(\begin{array}{cc} \frac{2}{5}\left(\frac{3+10}{5}\right)-{\frac{1}{5}}^{2} & 2\frac{1}{5}\\ 2\frac{1}{5} & \left[\left(\frac{10+2}{5}\right)\frac{3}{5}-{\frac{1}{5}}^{2}\right] \end{array}\right)$$

$$S_{\infty }^{-1}=\frac{1}{3+\frac{1}{10}}\left(\begin{array}{cc} \frac{2}{5}\left(\frac{13}{5}\right)-{\frac{1}{5}}^{2} & \frac{2}{5}\\ \frac{2}{5} & \left[\left(\frac{12}{5}\right)\frac{3}{5}-{\frac{1}{5}}^{2}\right] \end{array}\right)$$

$$S_{\infty }^{-1}=\frac{1}{15+\frac{1}{2}}\left(\begin{array}{cc} \frac{2}{5}\left(13\right)-\frac{1}{5} & 2\\ 2 & \left[\left(12\right)\frac{3}{5}-\frac{1}{5}\right] \end{array}\right)$$

$$S_{\infty }^{-1}=\frac{2}{31}\left(\begin{array}{cc} \frac{25}{5} & 2\\ 2 & \frac{35}{5} \end{array}\right)=\frac{2}{31}\left(\begin{array}{cc} 5 & 2\\ 2 & 7 \end{array}\right)=\frac{1}{31}\left(\begin{array}{cc} 10 & 4\\ 4 & 14 \end{array}\right),$$

whose determinant is:

$$S_{\infty }^{-1}=\frac{140-16}{31^{2}}=\frac{124}{31^{2}}=\frac{4}{31}.$$

Therefore, we have that:

$$S_{\infty }=\frac{31}{4}\times \frac{1}{31}\left(\begin{array}{cc} 14 & -4\\ -4 & 10 \end{array}\right)=\left(\begin{array}{cc} 7/2 & -1\\ -1 & 5/2 \end{array}\right),$$

for which:

$$\frac{\ln(|S_{\infty }|)}{2}=\frac{\ln(31/4)}{2}\approx 1.0238.$$

#### 3.1.3. Husimi Transform of Harmonic Groundstate

We will get the covariance matrix of the Gaussian associated with the Husimi transform of the harmonic ground state. Again, we have that:

$$S^{-1}=\frac{1}{d\left(1+as^{2}\right)+s^{2}\left(4\left[1+as^{2}\right]-b^{2}\right)}\left(\begin{array}{cc} a\left(d+4s^{2}\right)-b^{2} & 4bs^{2}\\ 4bs^{2} & 4s^{2}\left[\left(1+as^{2}\right)d-b^{2}s^{2}\right] \end{array}\right)$$

where:

$$\Sigma^{-1}=\left(\begin{array}{cc} \Sigma_{11}^{-1} & \Sigma_{12}^{-1}\\ \Sigma_{21}^{-1} & \Sigma_{22}^{-1} \end{array}\right)=\left(\begin{array}{cc} a & b\\ c & d \end{array}\right)$$

so for the inverse covariance matrix of the harmonic ground state, we have:

$$\Sigma_{0}^{-1}=\left(\begin{array}{cc} 2 & 0\\ 0 & 2 \end{array}\right)$$

since choosing a position domain of length L=1, we have that:

$$\Sigma_{0}=\left(\begin{array}{cc} 1/2 & 0\\ 0 & 1/2 \end{array}\right)$$

Choosing the parameter value $s=1/\sqrt{2}\Leftrightarrow 2s^{2}=1,$ then we have:

$$S_{0}^{-1}=\frac{1}{d+a+ad/2-b^{2}/2+2}\left(\begin{array}{cc} a\left(d+2\right)-b^{2} & 2b\\ 2b & \left[\left(2+a\right)d-b^{2}\right] \end{array}\right)$$

$$S_{0}^{-1}=\frac{1}{2+2+4/2-0+2}\left(\begin{array}{cc} 2(2+2)-0 & 0\\ 0 & \left[(2+2)2-0\right] \end{array}\right)$$

$$S_{0}^{-1}=\frac{1}{8}\left(\begin{array}{cc} 8 & 0\\ 0 & 8 \end{array}\right)=\left(\begin{array}{cc} 1 & 0\\ 0 & 1 \end{array}\right)=I_{d},$$

therefore, $S_{0}=I_{d}$ and $\det(S_{0})=1,$ for which:

$$\frac{\ln(|S_{0}|)}{2}=\frac{\ln(1)}{2}=0.$$

#### 3.1.4. Wehrl Entropy of a Gaussian State through Its Husimi Transform

Gaussian states in the Wigner formulation are represented simply by Gaussians. They are the only states for which their Wigner functions w(x,k)≥0 are nonnegative over all their domain. Moreover, the harmonic potential will transform Gaussian states into other Gaussian states until the steady-state solution is achieved [35].

We can evaluate their Wehrl entropy by the Husimi function h(x,k). The Husimi function, obtained by applying a Husimi transform to our Wigner function, will be another Gaussian since *w* is Gaussian, though in general different from w(x,k) in its analytical form. However, *h* will still be represented by a formula such as:

$$h\left(x,k,t\right)=\frac{1}{\sqrt{{|\Sigma |\left(2\pi \right)}^{d}}}\exp\left(-\frac{1}{2}\left(x,k\right)\Sigma^{-1}\left(\begin{array}{c} x\\ k \end{array}\right)\right)=\frac{1}{2\pi \sqrt{\left|\Sigma \right|}}\exp\left(-\frac{1}{2}\left(x,k\right)\Sigma^{-1}\left(\begin{array}{c} x\\ k \end{array}\right)\right),$$

where d=2.

The Wehrl’s entropy is obtained by calculating the following:

H=−∫hlog(h)dxdk,

$$H=-\int \frac{1}{2\pi \sqrt{\left|\Sigma \right|}}\exp\left(-\frac{1}{2}\left(x,k\right)\Sigma^{-1}\left(\begin{array}{c} x\\ k \end{array}\right)\right)\log\left(\frac{1}{2\pi \sqrt{\left|\Sigma \right|}}\exp\left(-\frac{1}{2}\left(x,k\right)\Sigma^{-1}\left(\begin{array}{c} x\\ k \end{array}\right)\right)\right)dxdk=$$

$$H=-\int \frac{1}{2\pi \sqrt{\left|\Sigma \right|}}\exp\left(-\frac{1}{2}\left(x,k\right)\Sigma^{-1}\left(\begin{array}{c} x\\ k \end{array}\right)\right)\left[\log\left(\frac{1}{2\pi \sqrt{\left|\Sigma \right|}}\right)-\frac{1}{2}\left(x,k\right)\Sigma^{-1}\left(\begin{array}{c} x\\ k \end{array}\right)\right]dxdk=$$

$$-\log\left(\frac{1}{2\pi \sqrt{\left|\Sigma \right|}}\right)\left[\frac{1}{2\pi \sqrt{\left|\Sigma \right|}}\int \exp\left(-\frac{1}{2}\left(x,k\right)\Sigma^{-1}\left(\begin{array}{c} x\\ k \end{array}\right)\right)dxdk\right]+$$

$$\frac{1}{2\pi \sqrt{\left|\Sigma \right|}}\int \exp\left(-\frac{1}{2}\left(x,k\right)\Sigma^{-1}\left(\begin{array}{c} x\\ k \end{array}\right)\right)\frac{1}{2}\left(x,k\right)\Sigma^{-1}\left(\begin{array}{c} x\\ k \end{array}\right)dxdk=$$

$$-\log\left(\frac{1}{2\pi \sqrt{\left|\Sigma \right|}}\right)+\frac{1/2}{2\pi \sqrt{\left|\Sigma \right|}}\int \exp\left(-\frac{1}{2}\left(x,k\right)\Sigma^{-1}\left(\begin{array}{c} x\\ k \end{array}\right)\right)\left(x,k\right)\Sigma^{-1}\left(\begin{array}{c} x\\ k \end{array}\right)dxdk,$$

since our Gaussians are normalized to 1. Since Σ is a positive definite matrix, this means it can be diagonalized into a matrix with positive eigenvalues:

$$\Sigma =Q^{-1}DQ,\;D=\mathrm{diag}\left(d_{i}\right),\;d_{i}>0,\;i\in 1,2.$$

Since then, its inverse $\Sigma^{-1}$ is also positive definite, one can perform the unique Cholesky factorization of $\Sigma^{-1}$ via a nonsingular upper triangular matrix *U*:

$$\Sigma^{-1}=U^{T}U,$$

where it holds that:

$$\left|\Sigma^{-1}\right|=\left|U^{T}U\right|={\left|U\right|}^{2}\Leftrightarrow \left|\Sigma \right|=1/{\left|U\right|}^{2},$$

so:

$$H=\log\left(2\pi \sqrt{\left|\Sigma \right|}\right)+\frac{1/2}{2\pi \sqrt{\left|\Sigma \right|}}\int \exp\left(-\frac{1}{2}\left(x,k\right)U^{T}U\left(\begin{array}{c} x\\ k \end{array}\right)\right)\left(x,k\right)U^{T}U\left(\begin{array}{c} x\\ k \end{array}\right)dxdk=$$

$$H=\log\left(\frac{2\pi }{\left|U\right|}\right)+\frac{|U|/2}{2\pi }\int \exp\left(-\frac{1}{2}\left(x,k\right)U^{T}U\left(\begin{array}{c} x\\ k \end{array}\right)\right)\left(x,k\right)U^{T}U\left(\begin{array}{c} x\\ k \end{array}\right)dxdk,$$

and defining the coordinate transformation:

$$\left(\begin{array}{c} y\\ z \end{array}\right)=U\left(\begin{array}{c} x\\ k \end{array}\right)\Leftrightarrow \left(y,z\right)=\left(x,k\right)U^{T},$$

which means that:

dydz=|U|dxdk⇔dxdk=dydz/|U|,

$$H=\log\left(\frac{2\pi }{\left|U\right|}\right)+\frac{|U|/2}{2\pi }\int \exp\left(-\frac{1}{2}\left(y,z\right)\left(\begin{array}{c} y\\ z \end{array}\right)\right)\left(y,z\right)\left(\begin{array}{c} y\\ z \end{array}\right)dydz/\left|U\right|=$$

$$H=\log\left(\frac{2\pi }{\left|U\right|}\right)+\frac{|U|/2}{2\pi }\int \exp\left(-\frac{y^{2}+z^{2}}{2}\right)\left(y^{2}+z^{2}\right)dydz/\left|U\right|=$$

$$H=\log\left(\frac{2\pi }{\left|U\right|}\right)+\frac{1/2}{2\pi }\int \exp\left(-\frac{y^{2}+z^{2}}{2}\right)\left(y^{2}+z^{2}\right)dydz=$$

$$H=\log\left(\frac{2\pi }{\left|U\right|}\right)+\frac{\langle y^{2}+z^{2}\rangle }{2},$$

one can notice that the second term is fixed, so it is a constant for all possible Gaussians, and therefore, the entropy is mostly defined by the first term. Since we have:

$$\left|\Sigma \right|=\frac{1}{{\left|U\right|}^{2}}\Rightarrow \sqrt{\left|\Sigma \right|}=\frac{1}{\left|U\right|},$$

then:

$$H=\log\left(2\pi \sqrt{\left|\Sigma \right|}\right)+\frac{\langle y^{2}+z^{2}\rangle }{2},$$

so, finally:

$$H=\frac{\log(|\Sigma |)}{2}+\log\left(2\pi \right)+\frac{\langle y^{2}+z^{2}\rangle }{2},$$

or equivalently:

$$H=\frac{\log(|\Sigma |)}{2}+C,\;C=\log\left(2\pi \right)+\frac{\langle y^{2}+z^{2}\rangle }{2},$$

which, after diagonalization of the covariance matrix, can be equivalently expressed as:

$$H=\frac{\log\left(d_{1}\right)+\log\left(d_{2}\right)}{2}+C,\;C=\log\left(2\pi \right)+\frac{\langle y^{2}+z^{2}\rangle }{2}.$$

Since solutions are known to converge to a steady state, this means that entropy will converge as well to its steady-state value. For the particular case of $D=I_{d},\;\gamma =1$, we have:

(15) $$\Sigma_{\infty }=\left(\begin{array}{cc} 3 & -1\\ -1 & 2 \end{array}\right),$$

To complete the calculation, we simply recall that:

$$\frac{\langle y^{2}+z^{2}\rangle }{2}=\frac{1/2}{2\pi }\int \exp\left(-\frac{y^{2}+z^{2}}{2}\right)\left(y^{2}+z^{2}\right)dydz=\frac{\frac{2}{2}}{2\pi }\int dz\exp\left(-\frac{z^{2}}{2}\right)\int dy\exp\left(-\frac{y^{2}}{2}\right)y^{2}=$$

$$\frac{1}{2\pi }\int dz\exp\left(-\frac{z^{2}}{2}\right)\left(-\int d\left[\exp\left(-\frac{y^{2}}{2}\right)\right]y\right)={\left[\frac{1}{\sqrt{2\pi }}\right]}^{2}\int dz\exp\left(-\frac{z^{2}}{2}\right)\int \exp\left(-\frac{y^{2}}{2}\right)dy=1,$$

However, because there is a constant shift by *C* in the value of the Wehrl entropy *H*, in our numerical results section, we will limit ourselves to report the nonconstant part of this entropy, namely $\frac{\log(|\Sigma |)}{2}$ for Gaussian states.

Given our knowledge of the convergence in the Wigner–Fokker–Planck equation to a Gaussian steady-state in an exponential decay fashion [3], we notice that, indeed, the entropy might not strictly increase (one has freedom to choose, mathematically, the variances of a Gaussian representing the Gaussian state as higher or lower than the respective ones for the steady-state solution). The expected behavior when starting from a Gaussian state as the initial condition is that, under a harmonic potential, the entropy will converge to the steady-state entropy value.

The advantage of having a numerical solver of open quantum systems in the Wigner–Fokker–Planck formulation is that we can monitor closely (up to numerical error) the evolution of the entropy functional we chose to observe (the Wehrl entropy in our case), observing its behavior to determine numerically the type of behavior (either monotonic or not) for a given set of initial conditions, though one knows that the entropy will converge to a steady-state value in an attractor fashion. We monitor the Wehrl entropy then for Gaussian states under a harmonic potential in the Wigner–Fokker–Planck model for open quantum systems via Monte Carlo numerics, solving our convection-diffusion equation as a stochastic equation with an Euler–Maruyama methodology, as described in the previous subsections.

#### 3.1.5. Numerical Results of Entropy Behavior

We present below the results of a numerical simulation of the Wigner–Fokker–Planck equation under a harmonic potential taking as initial condition a Gaussian state. In this case, the state is guaranteed to be Gaussian at all times, approaching in the limit as time goes to infinity for a known Gaussian steady-state analytical solution [3]. We specifically focus on the behavior of the Wehrl entropy (minus its constant shift) log(det(Σ))/2 applied to the Husimi transform of our Wigner function representing the Gaussian states taken as initial conditions. For reference, we include in our plots a straight line with the analytical value of the steady-state Wehrl entropy log(|Σ|)/2 to which the entropies for the numerical solutions are expected to converge (which was indeed observed in our numerical simulations, as our subsequent figures will show).

**Figure 1:**
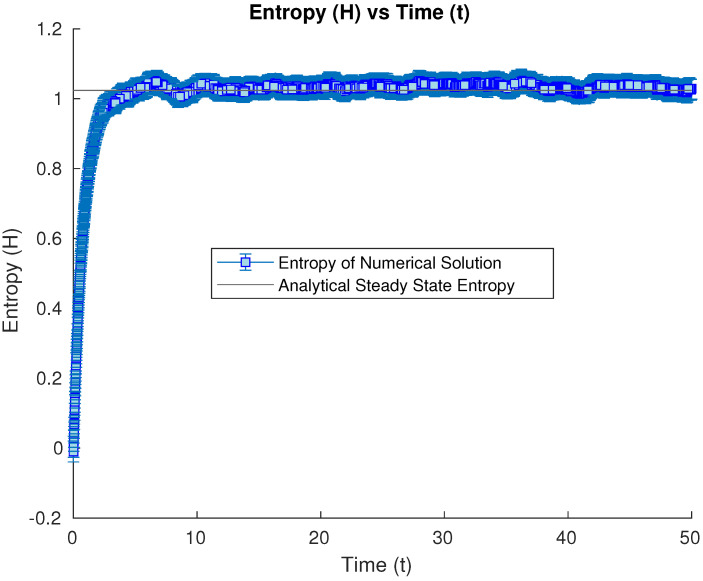
Numerical time evolution of the Wehrl entropy starting from a harmonic groundstate initial condition, until the steady state is achieved numerically.

Our first set of studies considers the evolution of an open quantum system whose initial condition is the harmonic ground state. After a long enough time, it converges numerically to the analytical steady-state solution. We study the numerical evolution of the Wehrl entropy at our different time iterations. In this case, the entropy increases from the initial value in the groundstate case towards the steady-state value, oscillating numerically around this limit value due to the inherent error in Monte Carlo methods (see Figure 1).

**Figure 2:**
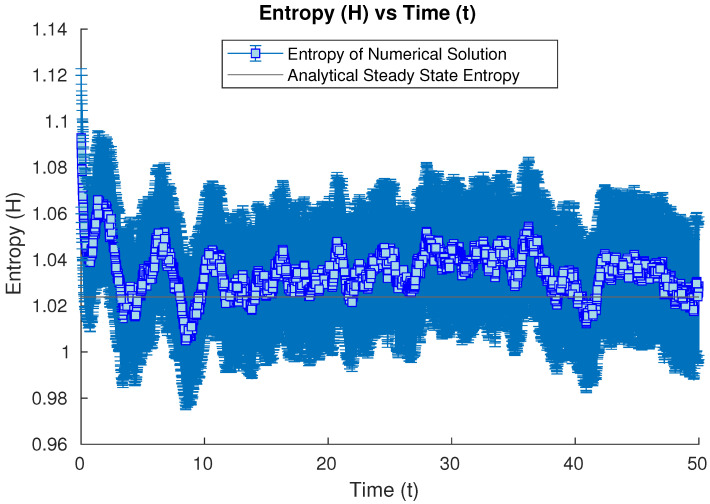
Numerical time evolution of the Wehrl entropy starting from a Gaussian state whose covariance is $2.25^{2}=5.0625$ times the harmonic groundstate one, until the steady state is achieved numerically. Error bars were included to also consider the inherent uncertainty of the Monte Carlo method in use.

**Figure 3:**
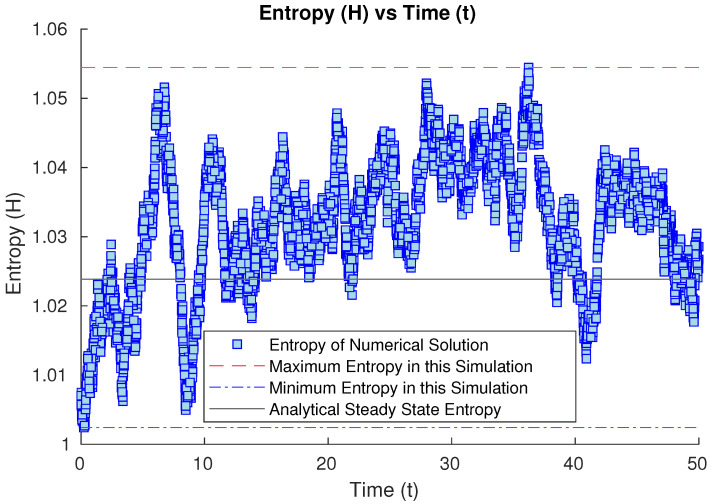
Numerical time evolution of the Wehrl entropy starting from the steady state as the initial condition, oscillating around it with the inherent numerical error of Monte Carlo methods.

Our second study considers the evolution of our open quantum system starting from a Gaussian state whose covariance matrix is $2.25^{2}=5.0625$ times the one for the harmonic oscillator. In this case, the entropy of the related Gaussian state is bigger than in the previous case, and it evolves by first sharply decreasing over a short time and then bouncing back up briefly (see Figure 2), until it decreases and oscillates numerically around the steady state. However, this brief nonmonotonic bounce is of the order of ΔH=0.02, which is below the inherent stochastic uncertainty of our Monte Carlo method (of the order of ϵ=0.03), obtained by observing the time evolution of our stochastic solver starting from the steady-state solution itself as initial condition (see Figure 3). Because the nonmonotonic bounce is below the uncertainty of the numerical method, up to the numerical error, we cannot deny the possibility of monotonic decay of the Wehrl’s entropy being present in this plot within the uncertainty bounds.

For the third set of results, we will try different cases where the initial condition is related to a Gaussian state, whose covariance matrix is not diagonal. We will try first as a sanity check to start with the steady state as the initial condition. This will render the numerical simulation as simply noisy oscillations around the known equilibrium state with its given entropy value (see Figure 3), and in fact, this simulation will indicate the inherent uncertainty of our Euler–Maruyama method as it oscillates numerically around the steady state with the stochastic error of the method.

**Figure 4:**
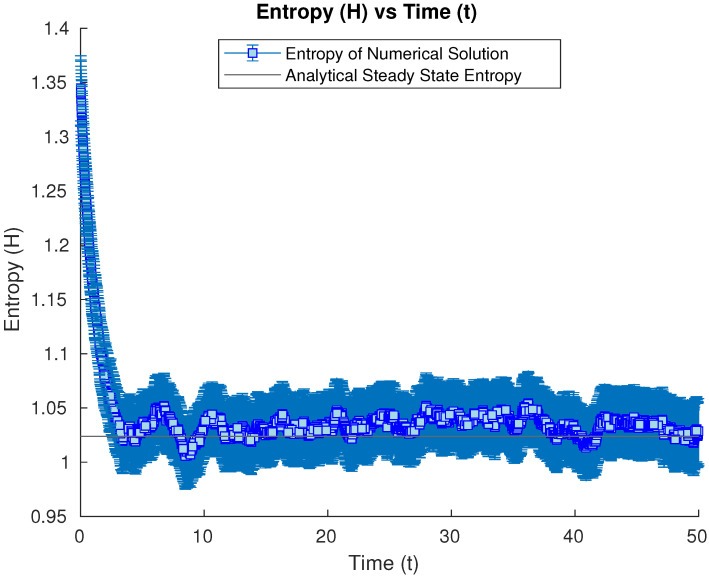
Numerical time evolution of the Wehrl entropy starting from a Gaussian state with a covariance matrix 1.5 times the steady-state one, having its entropy converge to the steady-state value.

We now will try as initial condition a Gaussian state, with the covariance matrix being a multiple of the steady state one, to study how the numerical solution will converge to the steady state (and particularly its entropy) from initial conditions with higher variances in the diagonalizing directions (more spread). We start then from a Gaussian state with a covariance matrix with values 1.5 times the ones of the steady state. Its entropy value is shown to converge to the steady-state entropy again (see Figure 4).

**Figure 5:**
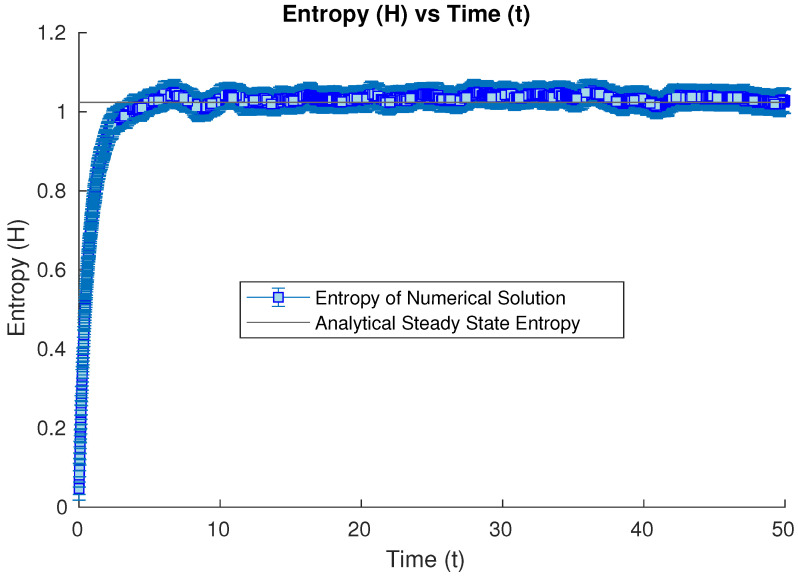
Numerical time evolution of the Wehrl entropy starting from a squeezed state such that $\Delta x=1=\sigma_{x},\;\Delta p=1/2=\sigma_{p}$, having its entropy converge to the steady-state value.

**Figure 6:**
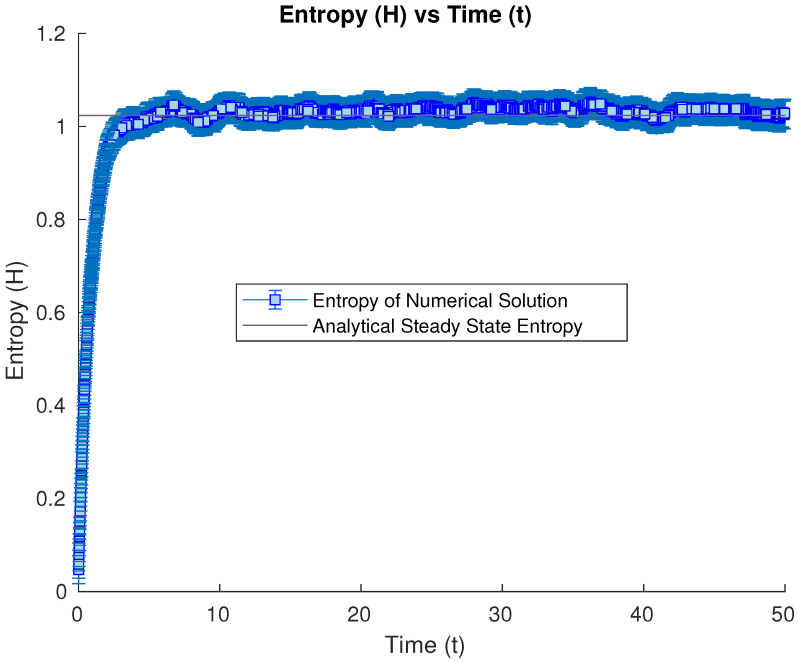
Numerical time evolution of the Wehrl entropy starting from a squeezed state such that $\Delta x=1/2=\sigma_{x},\;\Delta p=1=\sigma_{p}$, having its entropy converge to the steady-state value as well.

Our last numerical examples start from initial conditions reflecting squeezed states centered at the origin with diagonal covariance matrices. One of them is such that (in units where ¯h=1=m) $\Delta x=1=\sigma_{x},\;\Delta p=1/2=\sigma_{p}$ (Figure 5), and the other one is such that $\Delta x=1/2=\sigma_{x},\;\Delta p=1=\sigma_{p}$ (Figure 6). Its entropy values converge to the steady state one during the numerical evolution time for both cases (see Figure 5 and Figure 6).

## 4. Discussion

We find that the monotonic behavior of the entropy with time for the case with friction cannot be denied for any of the five initial conditions we picked. Without consideration of the inherent uncertainty in the numerical method of choice (Euler–Maruyama), one might feel tempted to interpret the inherent oscillations in the entropy evolution as nonmonotonic behavior. However, when including the natural stochastic error from the Euler–Maruyama-based method, one cannot disregard the possibility of monotonic behavior, since all oscillations are within the error bars of the numerical method. The neglect of these considerations would represent overfitting. Though we selected particular cases of Gaussian states as initial conditions, these were chosen based on their importance within the problem. Namely, the harmonic ground state is the paradigmatic case of a coherent state, studying as well a thermal state related to it, squeezed states are important as minimal uncertainty states with variances different from the coherent state ones, and the steady-state solution for the harmonic potential under noise is crucial to understand since all well-posed initial conditions converge to it. Though theoretical studies cannot guarantee monotonic behavior for a completely different entropy (the Von Neumann one), except for the frictionless case γ=0, the numerical evidence seems to suggest that at least for Gaussian states one should explore the theoretical possibility of monotonic behavior of the Wehrl entropy in this case (or trying to find a counterexample perhaps related to the case where the average behavior without uncertainty considerations seems monotonic on a naive analysis).

## 5. Conclusions

We studied numerically the behavior of the Wehrl entropy related to the quantum information of a benchmark open quantum system problem, formulated in terms of the Wigner–Fokker–Planck equation. This benchmark problem is namely the case of a harmonic potential subject to Markovian noise producing diffusion and friction over the quantum transport of the problem. The main motivation to study the quantum entropy for an open quantum system via Monte Carlo methods based on Euler–Maruyama techniques is the absence of theoretical results that can guarantee either monotonicity or its opposite of absolute entropies of importance such as the Von Neumann or the Wehrl one (except for the Von Neumann entropy in the particular frictionless case γ=0). Our numerical results for specific initial conditions, namely some particular Gaussian states related to the harmonic groundstate and the steady state (and some other Gaussian states with covariance matrices proportional to these two), seem to indicate that, within the error bounds of the numerical method, monotonicity could not be denied for the explored cases. These numerical results might motivate further theoretical work to study if one can guarantee for Gaussian states (or a subset of them) the monotonic behavior of Wehrl entropy in this benchmark case of a harmonic potential. On the other hand, one could also try to find a counterexample possibly related to the case that was closest yet unsuccessful (within the bounds of the numerical error of our method) to violating monotonicity from the particular cases of initial conditions selected. Further work will be considered in the extension of these numerical techniques for the case of non-Gaussian states or nonharmonic potentials, where the signed-particle method as in [11], for example, could be used.

## Data Availability

Code that produces the data supporting reported results can be found in the following GitHub repository: https://github.com/phjame/StochasticWFP (accessed on 29 February 2024).

## Appendix A. Weighted *L*_2_-Distance between Wigner Function and Steady-State Solution for Harmonic Potential

We consider the following weighted $L_{2}$-distance between the Wigner function w(x,p,t) at time *t* and the steady-state solution for the harmonic potential $\mu \left(x,p\right)=\lim_{t\to \infty }w\left(x,p,t\right)$ as in [2]:

(A1) $$||\frac{w-\mu }{\sqrt{\mu }}|{|}_{L^{2}}.$$

If we proceed to calculate this weighted distance, we have that:

(A2) $$\begin{array}{cc} ||\frac{w-\mu }{\sqrt{\mu }}|{|}_{L^{2}}\; & =\int dxdk{\left(w-\mu \right)}^{2}/\mu \\ \; & =\int dxdk(w^{2}/\mu -2w+\mu )\\ \; & =\int dxdk(w^{2}/\mu )-1, \end{array}$$

the last line standing true because Wigner functions are normalized to 1 in phase space. Now, the integral in Equation (A2) will only converge if the resulting function $w^{2}/\mu$ is indeed a Gaussian distribution. If the subtraction of the inverse covariance matrices of $w^{2}$ and μ is not positive definite, then the related integral will diverge and the norm will blow up to infinity. This imposes constraints as to which Wigner functions will result in this $L_{2}$-distance being well defined. Specifically, let us take μ to be the normalized Gaussian centered at the origin with covariance matrix:

(A3) $$\Sigma_{\mu }=\left[\begin{array}{cc} 3 & -1\\ -1 & 2 \end{array}\right],$$

and *w* to have the covariance matrix:

(A4) $$\Sigma_{w}\left(t\right)=\left[\begin{array}{cc} A(t) & B(t)\\ B(t) & D(t) \end{array}\right],$$

where A(t), B(t), and D(t) are time-dependent parameters such that not only $\Sigma_{w}$ is positive definite at all times but also that $w^{2}/\mu$ has a positive definite covariance matrix at all times. This is satisfied for $w^{2}/\mu$ if the condition $\det\left(2\Sigma_{w}^{-1}-\Sigma_{\mu }^{-1}\right)\left(t\right)>0$ holds. One can show that this condition is equivalent to satisfying:

(A5) $$\begin{array}{c} Q\equiv \frac{20-4B-B^{2}+A\left(D-4\right)-6D}{-6B^{2}+A\left(6D-20\right)}<0. \end{array}$$

In the case where the initial condition ${w|}_{t=0}$ is the harmonic oscillator ground state, the norm of ${w|}_{t=0}=w_{0}$ is well defined. The covariance matrix of $w_{0}$ is, after taking ¯h=m=ω=1, $\Sigma_{0}=I/2$, where *I* is the identity matrix. Thus, Q=−61/34 for $w_{0}$, which is less than 0.

Now, let us consider the Wigner functions with covariance matrices $C_{0}\Sigma_{0}$ and $C_{\mu }\Sigma_{\mu }$, where $C_{0}$ and $C_{\mu }$ are positive real numbers. There exist valid values of $C_{0}$ and $C_{\mu }$ such that the norm (A1) is defined. Applying the condition (A5) to these covariance matrices, we obtain the following results:

(A6) $$\begin{array}{cc} \; & C_{0}\in \left(0,10-2\sqrt{5}\right)\cup \left(\frac{20}{3},10+2\sqrt{5}\right)), \end{array}$$

(A7) $$\begin{array}{cc} \; & C_{\mu }<2. \end{array}$$

These conditions are part of the motivations in our choice of coefficients in the analysis of the Wehrl entropy for our Monte Carlo numerical solutions presented in this work.
